# Removing Journal Impact Factors

**DOI:** 10.1128/mBio.00185-17

**Published:** 2017-02-21

**Authors:** Richard J. Lamont

**Affiliations:** Oral Immunology and Infectious Diseases, University of Louisville, Louisville, Kentucky, USA

## LETTER

I applaud the ASM for the decision to put their head above the parapet and cast aside the essentially meaningless metric of journal impact factor (IF). What began as a measure intended to allow quantitative evaluation of the importance of journals, IFs have virtually consumed all other (often more relevant) considerations regarding quality of publication. Ironically, for many of the scientists who have perpetuated this system, the consequences have amounted to a rod for their own backs. IFs have created a faux exclusivity in the journals that have successfully gamed the system, and IFs are commonly used in faculty evaluations. As a journal editor and departmental chair, I have been equally complicit in the misuse of IFs; to do otherwise has seemed pointless. However, by taking the first steps to apply the brakes to the IF runaway juggernaut, the ASM has provided an important precedent for curtailing the encroachment of IFs. I have used the ASM’s stance to justify no longer considering IFs in the evaluation of faculty in my department. Perhaps now the process that gained momentum by a positive-feedback loop can be undone by a negative-feedback loop.

